# Chloral in Toothache

**Published:** 1872-01

**Authors:** 


*Chloral in Toothache.*—In the "British Medical Journal," Dr. David Page states that he has been for some time in the habit of using *chloral hydrate*, not only as an internal sedative in cases of severe dental neuralgia and of caries where it was at the time inadvisable to permit extraction, but more frequently as a direct local application to the carious tooth. A few grains of the solid hydrate introduced into the cavily of the tooth upon the point of a quill, speedily dissolve there; and in the course of a few minutes, during which a not unpleasant warm sensation is experienced, the pain is either deadened, or more often effectually allayed. A second or third application may be resorted to if necessary. The hours of agony that various circumstances may otherwise entail, are in this way avoided; and in the last instance under my notice, a young lady in a delicate state of health, suffering intensely from a carious tooth, received immediate relief from this application.

*Dentistry and its Difficulties.*—In the "London Lancet," we find the following report of a paper on Dental Pathology: "The difficulties and accidents of dental surgery are very ably and candidly discussed in a paper by Mr. W. A. N. Cattlin, F.R.C.S., reprinted from the "Transactions of the Odontological Society of Great Britain." Mr. Cattlin speaks from a very large experience. He has published with his paper five sheets of illustrations of specimens of diseased teeth, from the collections of Mr. Cartwright and Mr. Tomes, and from his own. The way in which they have been photographed in the first instance and again taken from the photographs is very effective. They supply quite a large and beautiful illustration of the diseases of teeth and the difficulties of the dentist. Mr. Cattlin's remarks on peculiar positions of the wisdom teeth, on the danger of producing fracture of the tuberosity of the superior maxilla in extracting the third upper molars, and on the steps to be taken to avoid fracturing the floor of the antrum in drawing teeth in this neighbourhood which offer any peculiar resistance, are worthy of attention, as also are his criticisms of various recent instruments and operative precedures. A peculiar case is related of a diseased soft state of the alveolar processs in which canaliculi were absent. Mr. Cattlin, while a thorough specialist, brings to the elucidation of his subject light from all quarters, and speaks at once like a physician and physiologist in treating of dental hæmorrhage, of the various reflex effects of bad teeth, sometimes much overlooked, and of the subject of anæsthesia, in which dentists have an unusal experience. Mr. Cattlin has studied the relation of asphyxia to anæsthesia. In his former experiments he failed to produce anæsthesia by simple suffocation brought about by the reinspiration of the same air. But, making a similar experiment lately, and carrying the asphyxia a step further, he has succeeded in producing perfect anæsthesia, under which the animal was operated on, and from which it perfectly recovered."

*Removal of a Fibrous Tumour from the Roof of the Mouth.*—In the same journal the following operations are described:

The patient was a woman of about twenty years of age. From the surface of the hard palate, to the left of the median line, there sprang a tumour of the size of a large filbert, somewhat lobulated, and covered with healthy mucous membrane. As the roof of the mouth was rather low, this tumour sufficed to fill the entire cavity of the closed mouth. An incision having been made in the mucous membrane, the tumour was readily turned out by means of an elevator, and proved, as had been suspected, to be of the fibrous variety.

